# Gene expression through comparative transcriptome analysis unravels the molecular mechanisms of fertilizer-induced hormonal regulation and leaf senescence in maize for enhanced yield in semiarid regions

**DOI:** 10.1186/s12870-025-06303-5

**Published:** 2025-03-13

**Authors:** Setor Kwami Fudjoe, Shangli Shi, Lingling Li, Sumera Anwar, Junhong Xie, Francis Chimsah, Linlin Wang

**Affiliations:** 1 State Key Laboratory of Aridland Crop Science, Gansu Agricultural University, Lanzhou, 730070 China; 2 College of Grassland Science, Gansu Agricultural University, Lanzhou, 730070 China; 3 College of Agronomy, Gansu Agricultural University, Lanzhou, 730070 China; 4 Department of Botany, Government College Women University Faisalabad, Faisalabad, 38000 Pakistan; 5 Faculty of Agriculture, Food and Consumer Science, University for Development Studies, Tamale, Ghana

**Keywords:** Maize, Transcriptome sequencing, Physiological analysis, Photosynthesis, Transcription factor, Plant hormone

## Abstract

**Aims:**

Fertilizers can significantly influence leaf senescence and hormonal regulation, which in turn impacts crop yield. Despite significant advancements in understanding fertilizer effects on plant growth, the specific molecular mechanisms through which fertilizers influence hormonal regulation and leaf senescence, and subsequent impact on yield, remain underexplored. This study addresses this critical gap by examining transcriptional, physiological, and molecular mechanisms in the semiarid regions of rainfed spring maize under long-term fertilizers.

**Methods:**

Fertilizer treatments include no amendment (NA), inorganic fertilizer (CF), combined inorganic and organic fertilizer (SC), organic fertilizer (SM), and maize straw (MS) replicated three times.

**Results:**

The highest number of differentially expressed genes (DEGs) were observed under CF (3972) followed by SC (1949) in comparison to NA, showing a strong effect of inorganic fertilizer on gene expressions. The Kyoto Encyclopedia of Genes and Genomes (KEGG) analysis revealed that numerous genes involved in the biosynthesis of secondary metabolites, plant hormone signaling, photosynthesis pathways, and metabolic pathways showed varied expressions of up- and downregulation. Genes involved in the ethylene, abscisic acid, jasmonic acid, salicylic acid, and brassinosteroid pathways indicated their interaction and promoted leaf senescence, whereas those related to auxin and gibberellin pathways had minimal impact. In the ethylene pathway known to influence senescence, two ethylene receptor (ETR) genes (Zm00001d013486 and Zm00001d021687) were downregulated, whereas, two ethylene-insensitive proteins 3 (EIN2) genes (Zm00001d053594 and Zm00001d033625) showed upregulation in the CF, SC and SM treatments. Furthermore, 86 highly up-regulated genes involved in the photosynthesis pathway encompassing components such as photosynthesis antenna, photosynthesis complexes II, cytochrome complexes, photosynthesis electron transport, and ATP complex in SC and CF compared to SM and MS.

**Conclusion:**

In summary, the study finds that DEGs showed stronger responses to inorganic fertilizers, likely due to organic fertilizers decomposing at a slower rate. Nevertheless, transcriptional and physiological analyses indicate that the SC treatment sustainably enhances maize productivity without causing adverse environmental effects, outperforming the other treatments (NA, CF, SM, MS). These results provide new perspectives on genetic regulation and pathway discovery in rainfed maize cultivation in semiarid areas.

**Supplementary Information:**

The online version contains supplementary material available at 10.1186/s12870-025-06303-5.

## Introduction

Throughout the life cycle of leaves, they experience three developmental phases: an increasing functionality phase during early growth, a full functionality phase at maturity, and a decreasing functionality phase during senescence. The senescence process is genetically regulated, involving significant changes in gene expression that lead to cell degradation and the reallocation of nutrients to new organs [1, 2]. Senescence-associated genes (SAGs) play a crucial role in protein degradation and nitrogen recycling. Although senescence is a form of programmed cell death, it is vital for plant fitness as it helps remobilize nutrients to growing and reproductive organs. This process is influenced by internal factors like age, hormones and environmental factors such as drought and UV-B radiation under different fertilization systems [3, 4]. During senescence, numerous organic, cellular, and molecular changes occur in a highly coordinated manner. The yellowing of senescing leaves is linked to fertilization biochemical changes, including chlorophyll loss, protein and RNA degradation, and reduced photosynthetic activity. Accelerated leaf senescence reduces carbon assimilation, thereby decreasing plant growth and yield via urea translocation. Earlier research has identified and characterized SAGs and senescence-related mutants in plants like Arabidopsis, reveal the complex regulation of gene expression during senescence [5]. At the onset of senescence, a subset of SAGs is up-regulated, while most genes expressed in non-senescent leaves, including those related to photosynthesis, are down-regulated. Since photosynthesis is a significant source of dry matter production, it is closely related to yield formation [6].

Plant hormones significantly influence all stages of leaf senescence: initiation, progression, and terminal phase. Ethylene (ETH), jasmonic acid (JA), salicylic acid (SA), and abscisic acid (ABA) promote senescence, while cytokinins (CKs), gibberellic acid (GA), and auxin (AUX) delay it. Senescence is accelerated by ethylene, ABA, and JA, which also mediate responses to various stresses under fertilization [7, 8]. Environmental factors like nutrient deficiency, drought, salt, temperature extremes, darkness, pathogen infection, and insect feeding are critical in regulating senescence. Exogenous ethylene enhances leaf yellowing, and ethylene biosynthesis genes are differentially expressed in senescing leaves [9, 10]. Ethylene-insensitive mutants show delayed senescence. ABA and methyl jasmonic acid also accelerate senescence, and JA-insensitive mutants do not display JA-dependent senescence. Increased cytokinin levels are associated with delayed leaf ageing. In contrast, the overproduction of cytokinins in plants such as tobacco, petunia, cassava, and lettuce has been demonstrated to postpone leaf senescence under low nitrogen nutrition [11, 12]. Leaf senescence involves the upregulation of SAGs, including genes encoding catabolic enzymes and transcription factors, with hormones like ABA, JA, and ETH inducing SAGs and cytokinins reducing their expression [13, 14].

Transcription factors (TFs) regulate gene expression by stimulating or subduing target genes and are crucial in hormone signaling, stress responses, metabolism, and leaf senescence. Key groups of senescence-related TFs include the NAC, WRKY, MYB, C2H2 zinc-finger, bZIP, and AP2/EREBP families [15–17]. Previous research using Maize and rice identified WRKY TFs such as WRKY7, WRKY18, WRKY42, and WRKY69, which regulate plant defence and senescence. In Maize, the NAC family, particularly NAC092/AtNAC2/ORESARA1 (ORE1), is significant for regulating senescence, with mutants of ORE1 delaying leaf senescence [18]. Overexpression of ORE1 significantly up-regulates 78 senescence-associated genes (SAGs) downstream. The NAC family is one of the largest TF families in plants, with about 106 members in Maize and Arabidopsis involved in development and stress responses, which also regulate senescence in many plant species [19–21].

Maize is vital for global food security, with China being a leading producer, contributing about 31% of global production. The Loess Plateau in China, is a key rainfed agricultural region, which accounts for 17.9% of the nation's crop production area. Nutrient deficiencies and insufficient rainfall are major factors limiting maize yield in this region [22–24]. Effective fertilization, particularly in arid and semiarid areas with limited soil organic matter, is crucial for maize growth. In semiarid regions, a strategic approach to managing natural resources and using inorganic fertilizers judiciously can improve crop yields and reduce yield fluctuations. Essential nutrients for better yields include nitrogen, phosphorus, and potassium. In arid regions, combining organic with minimal inorganic fertilizers can enhance drought resilience. Sustainable approaches that maximize crop yield while minimizing reliance on inorganic fertilizers are needed to protect the environment and reduce production costs [24–26].

This study investigated the comparative transcriptome elements and molecular mechanisms associated with plant hormone and leaf senescence in maize, focusing on the effects of inorganic and organic fertilization in the semiarid regions. The aim was to identify molecular regulatory dynamics and gene expression patterns that influence plant hormone as well as leaf senescence which affect grain filling, and could be used in future breeding programs and genetic modifications to improve maize yield. By profiling genomic variations in maize leaves under different fertilization practices, the study recognized key structural genes, transcription factors, and signaling paths involved in senescence. The research utilized RNA sequencing (RNA-Seq) analysis during the flowering stage, revealing differentially expressed genes and enriched biological pathways through Gene Ontology (GO), Kyoto Encyclopedia of Genes and Genomes (KEGG), and DEG analysis. Understanding these transcriptional mechanisms can help provide essential data for enhancing nutrient input for maize productivity, particularly in the semiarid Loess Plateau region of China.

## Materials and methods

### Description of experimental area

The field experiment of maize and the application of fertilizer treatments were started in 2012, however, the analysis and data collection were performed in 2023 cropping seasons. The field area was located at the Rainfed Agricultural Experimental station of the Gansu Agricultural University (35°28′N, 104°44′E, elevation: 1971 m above sea level), located in Gansu Province, NW China. The study area has a semiarid environment with an annual frost-free period of 140 days and an average elevation of 2,000 m. The semiarid Loess Plateau region has a steep terrain prone to erosion. The soil in the region is aeolian and locally known as Huangmian with ≥ 50% sand, having loamy textured Chinese Soil Taxonomy Cooperative Research Group [27], and classified as Calcaric Cambisol according to the by the FAO [28]. The soil physiochemical properties at the experimental site before sowing in 2023 are shown in Supplementary Table S1.

Long–term climatic records also show the yearly evaporation rate was 1531 mm, and the average annual radiation was 5930 MJ m-2. The average annual growing season precipitation and mean temperature at the site, with about 60% of that rainfall falling between July and September. From January to August each year, the temperature fluctuated between -23°C and 37°C, and the average annual precipitation was 390.8 mm.

### Description of treatments

The experiment was a randomized complete block design with five practices and three replicates per treatment. Thus, the study included five treatments, namely: (i) no fertilizer (NA); (ii) chemical fertilizer (CF) contained 200 kgNha^−1^ of urea and 150 kg P_2_O_5_ ha^−1^ of triple superphosphate; (iii) inorganic fertilizer plus organic fertilizer (SC) contained 3.03 tha^−1^ of organic fertilizer, 100 kgNha^−1^ of urea, and 120 kg P_2_O_5_ ha^−1^ of triple superphosphate; (iv) Organic fertilizer (SM) contained 6.06 tha-1 of organic fertilizer, and 90 kg P_2_O_5_ ha^−1^ of triple superphosphate; and (v) maize straw (MS) contained 28.5 tha^−1^ combined with triple superphosphate of 36 kgha^−1^. The treatments were replicated 3 times, giving a total of 15 plots, each measuring an area of 3 m × 14.2 m. Each experimental unit had an area of 110 cm with narrow ridges 15 cm high × 40 cm long and alternating with ridges 10 cm high × 70 cm wide (Supplementary Figure S1 and S2). Treatments SC, SM, and MS were applied at the same input. Urea, organic fertilizer, and maize straw were broadcasted and integrated into the top 20 cm soil layer. Samples of maize straw and organic fertilizer obtained during the application were collected and analyzed for nutrient concentration using an Elementar vario MACRO cube (Elementar, Hanau, Germany), as listed in Supplementary Table S2.

### Field layout

The source of each plant seed and planting material was purchased from (Gansu Daxing Agricultural Technology Co., Gansu, China). Glyphosate (30%) herbicide was applied to control weeds in the plots before planting. The ridge-furrow sowing system was adopted with plastic mulching (Supplementary Figure S2). Field layout comprised of alternating wide and narrow ridges with the narrow ridges having a height of 15 cm and a width of 0.40 cm, while the wide ridges are slightly lower, with a height of 10 cm and a width of 70 cm. The distance between adjacent ridges were 110 cm. Holes were made in the plastic mulch within the furrows, and maize seeds of cultivar Pioneer 335 were sown into these holes at a density of 52,500 plants ha^−1^ in late April. This sowing method likely aids in moisture retention, and weed suppression, regulates temperature around the seeds, and promotes better growth conditions for maize in the experiment. The maize crop was harvested in late September. Pests and diseases were observed and controlled according to the conventional practices in the study area. Weeding was also done manually intermittently by hand during the season when needed.

Soil pH was measured using a 1:2.5 soil-to-water ratio, total nitrogen (TN) via the Kjeldahl method, Olsen phosphorus through sodium bicarbonate extraction, and soil organic carbon (SOC) by the Walkley–Black wet oxidation method [29, 30].

### Measurement of plant physiological indices

The flag leaves were harvested at the flowering stage and promptly frozen in liquid nitrogen to minimize biological variability. To ensure uniformity, plant material was gathered randomly from at least six plants, and composite samples were created. Similar to the grain samples, the flag leaf samples were divided into two portions, with the first portion frozen in liquid nitrogen on-site, transported back to the laboratory, and stored at -80℃ for RNA and hormone analysis.

The second portion of flag leaves was weighed using an electronic balance in the field to assess their fresh weight. The dry weight of the flag leaves was determined by oven-drying the samples at 80°C until a constant weight was achieved [30]. Grain yields were determined by oven-drying at 105°C for 35 min, followed by further drying to a constant weight at 83°C and subsequent reweighing [31]. The yields of flag leaves were then extrapolated to kilograms per hectare.

Aboveground plant parts (flag leaves) were sampled from each plot to measure nitrogen accumulation at the flowering stage. The Maize leaves samples are cleaned, dried, and then ground or homogenized into a fine powder using a ball mill or similar equipment. The total nitrogen (TN) content was determined using the Kjeldahl method [32]. The ground maize samples are treated with hydrochloric acid (HCl) to remove any inorganic carbon present, such as carbonates. This step ensures that the measured OC content represents only the organic carbon fraction. The OC content in the maize leave samples is determined using the dry combustion method [33].

The physiological changes in maize seed tissue under various fertilization treatments (CF, SC, SM, and MS) compared to no fertilizer (NA) were analyzed in triplicate. Levels of malondialdehyde (MDA) and hydrogen peroxide (H_2_O_2_) were measured using Solarbio test kits as outlined by Wang et al. [6]. While the antioxidant enzyme activities comprising of superoxide dismutase (SOD), peroxidase (POD), catalase (CAT), and ascorbate peroxidase (APX), were assessed using kits from Jiangsu Meibiao Biotechnology Co., Ltd. (Yancheng, China).

### RNA extraction

Total RNA was isolated from the leaves tissue utilizing TRIzol® Reagent following the manufacturer's instructions (Invitrogen), with genomic DNA removal accomplished using DNase I (TaKara). Approximately 5 to 8 mg total RNA from each sample based on the 5 treatment (NA, CF, SC, SM and MS) replicated three times was used to construct RNA-Seq libraries. Subsequently, RNA quality was assessed using the 2100 Bioanalyser (Agilent) and quantified using the ND-2000 (NanoDrop Technologies). Only high-quality RNA samples among the 5 treatments meeting the criteria (OD260/280 = 1.8 ~ 2.2, OD260/230 ≥ 2.0, RIN ≥ 6.5, 28S:18S ≥ 1.0, > 10 μg) were employed for constructing the sequencing library.

### Library preparation and Illumina Hiseq sequencing

Transcriptome libraries for RNA-seq were prepared utilizing the TruSeqTM RNA sample preparation Kit from Illumina (San Diego, CA), with 1 μg of total RNA employed among the five treatments (NA, CF, SC, SM and MS) replicated three times. Initially, messenger RNA was isolated via polyA selection using oligo(dT) beads and then fragmented with a fragmentation buffer. Subsequent steps, including cDNA synthesis, end repair, A-base addition, and ligation of Illumina-indexed adaptors, were conducted following Illumina's protocol. The libraries were size-selected for cDNA fragments of 200–300 bp on 2% Low Range Ultra Agarose and then subjected to PCR amplification using Phusion DNA polymerase (NEB) for 15 PCR cycles. Following quantification using TBS380, the paired-end libraries were sequenced using Illumina NovaSeq 6000 sequencing (150 bp*2, Shanghai BIOZERON Co., Ltd).

### Reads quality control and mapping

The initial paired-end reads underwent trimming and quality control using Trimmomatic, applying the parameters (SLIDINGWINDOW:4:15 MINLEN:75) (version 0.36 available at http://www.usadellab.org/cms/uploads/supplementary/Trimmomatic). Subsequently, the resulting clean reads were individually aligned to the reference genome in orientation mode using hisat2 software (https://ccb.jhu.edu/software/hisat2/index.shtml) with default settings. The quality of these data was assessed using qualimap_v2.21 (http://qualimap.bioinfo.cipf.es/). Gene reads were quantified using htseq (https://htseq.readthedocs.io/en/release_0.11.1/). The sequencing data from this study have been deposited at the NCBI under BioProject Accession ID PRJNA1110623.

### Differential expression analysis and functional enrichment

To categorize differential expression genes (DEGs) between the leaf tissue samples based on the 5 treatment (NA, CF, SC, SM and MS) replicated three times, the expression level of each gene was assessed using the fragments per kilobase of exon per million mapped reads (FRKM) method. The R statistical package edgeR (Empirical Analysis of Digital Gene Expression in R, available at http://www.bioconductor.org/packages/release/bioc/html/edgeR.html/) was employed for the differential expression analysis. DEGs were determined based on specific criteria: a logarithmic fold change greater than 2 and a false discovery rate (FDR) below 0.05. To elucidate the functions of these differentially expressed genes, GO functional enrichment and KEGG pathway analysis were conducted using Goatools (https://github.com/tanghaibao/Goatools) and KOBAS (http://kobas.cbi.pku.edu.cn/home.do). DEGs were considered significantly enriched in GO terms and metabolic pathways if their Bonferroni-corrected *P*-value was less than 0.05. Additionally, all genes were queried against the plantTFDB (http://planttfdb.cbi.edu.cn) with a cutoff E-value of ≤ 1e^−5^ to ascertain putative transcription factors (TFs).

### Quantitative real-time PCR

We conducted further validation of RNA-seq results for 12 randomly selected DEGs associated with maize leaf tissue development using quantitative RT-PCR using SYBR Green I (Bio-Rad) and a CFX96 real-time PCR detection system (Bio-Rad, Hercules, USA). Each RNA sample underwent quantitative analysis a minimum of three times. The amplification conditions included an initial denaturation at 95 ◦C for 15 min, followed by 40 cycles of denaturation at 95 ◦C for 10 s, annealing at 60 ◦C for 20 s, and extension at 72 ◦C for 30 s. Additionally, a melting curve program (95 ◦C for 10 s followed by a gradual increase from 65 ◦C to 95 ◦C in 5-s increments of 0.5 ◦C) was employed to confirm the specificity of the PCR amplification. The relative expression levels were calculated using the method described by Livak and Schmittgen [34]. Gene-specific primers for amplifying fragments corresponding to the selected genes were designed using the NCBI Primer-BLAST tool (Supplementary Table S3). We utilized gene ID Zm00001d021288 as an internal control which was used as house-keeping gene to standardize the expression level of the target genes.

### Statistical analysis

Data analysis was performed using one-way ANOVA. The significant distinct means among treatments were determined using a Duncan test at a significance level of 0.05, conducted with SPSS version 22 (IBM Corporation, Chicago, USA, 2013). Graphs were generated using GraphPad Prism v18 2019.

## Results

### Summary of differentially expressed genes (DEG) under Fertilization practices

**Fig. 1:**
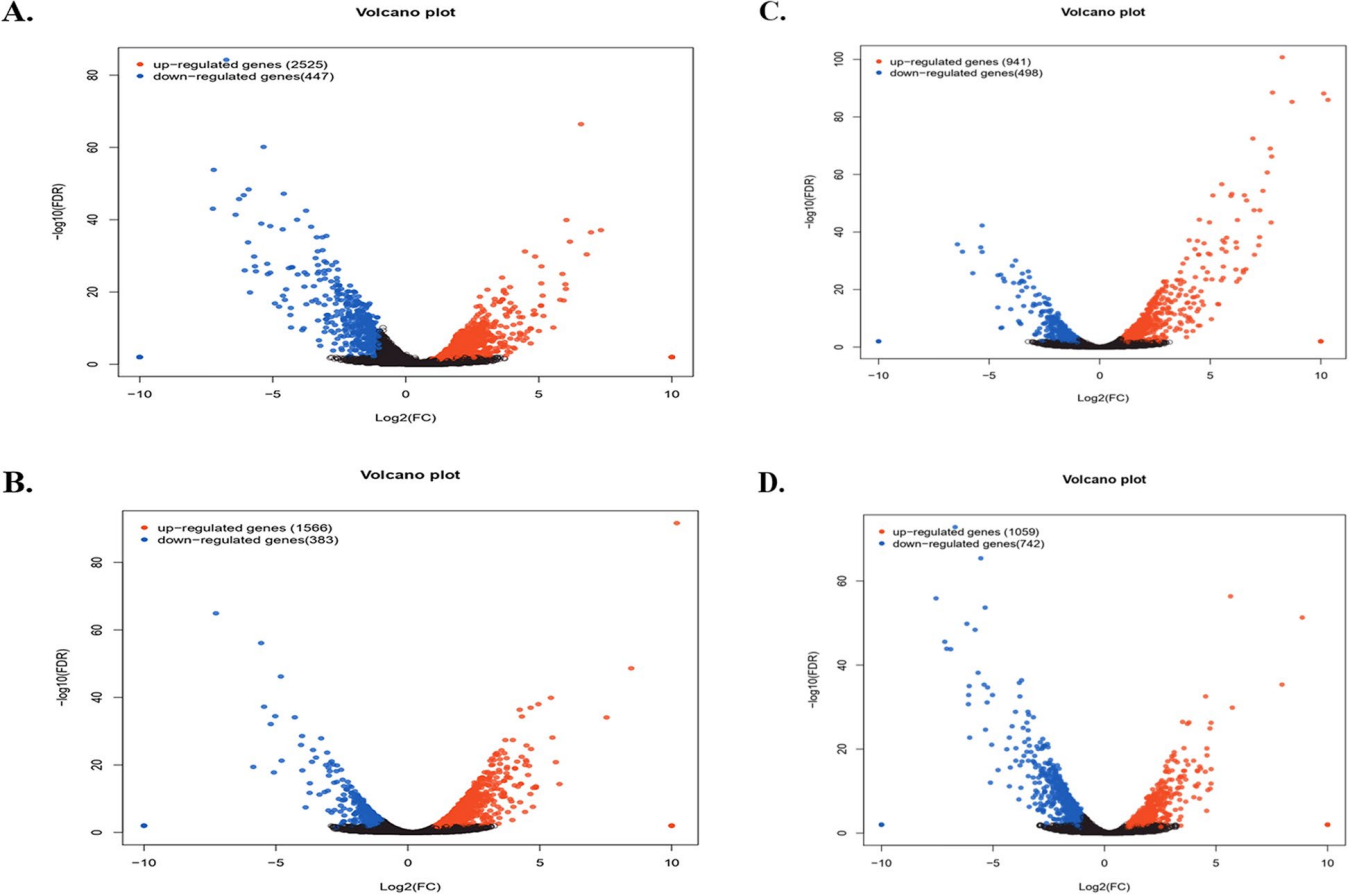
Volcano plot of gene expression level in CF vs NA (**A**), SC vs NA (**B**), SM vs NA (**C**), and MS vs NA (**D**). Red dots indicate up-regulated genes and blue dots indicate downregulated genes in differentially expressed genes (DEGs). NA, No fertilization; CF, inorganic fertilizer; SC, inorganic fertilizer plus commercial organic fertilizer; SC, commercial organic fertilizer; MS, maize straw

Using the criteria of |fold changes|> 1 and *P*-value < 0.05, a total of 8,161 DEGs (6,091 up-regulated and 2,070 downregulated) were identified across the four fertilization treatments and successfully annotated in a public database. Comparative analysis showed that 4,921 genes exhibited the highest DEGs in CF vs. NA compared to SC vs. NA in leaf samples, with 4,091 up-regulated and 830 down-regulated. Additionally, 1,439 and 1,801 genes were uniquely co-expressed under SM vs. NA and MS vs. NA treatments, respectively (Fig. 1A-D).

### Gene Ontology (GO) and Kyoto Encyclopedia of Genes and Genomes (KEEG) functional annotation enrichment analysis of DEGs under fertilization treatments

To investigate maize functions, GO annotation of leaf samples under different fertilization treatments was conducted to identify the genetic functions or paths most associated with DEGs. GO analysis revealed significant differences in biological processes (BP), molecular functions (MF), and cellular components (CC). The GO terms linked to the DEGs are shown in Supplementary Fig. S3. In the comparisons of CF vs. NA, SC vs. NA, SM vs. NA, and MS vs. NA, enriched GO terms included cellular process, metabolic process, and biological regulation (BP); binding and catalytic activity (MF); and cellular, anatomical entity and protein-containing complex (CC) (Supplementary Fig. S3). The highest proportion of up-regulated and downregulated genes involved in MF activity was observed in CF vs. NA, SC vs. NA, SM vs. NA, and MS vs. NA, compared to CC and BP activities. The CF vs. NA and SM vs. NA comparisons had more up-regulated genes in MF, CC, and BP, while SC vs. NA and MS vs. NA showed higher numbers of downregulated genes in the CC category (Supplementary Fig. S3).

**Fig. 2:**
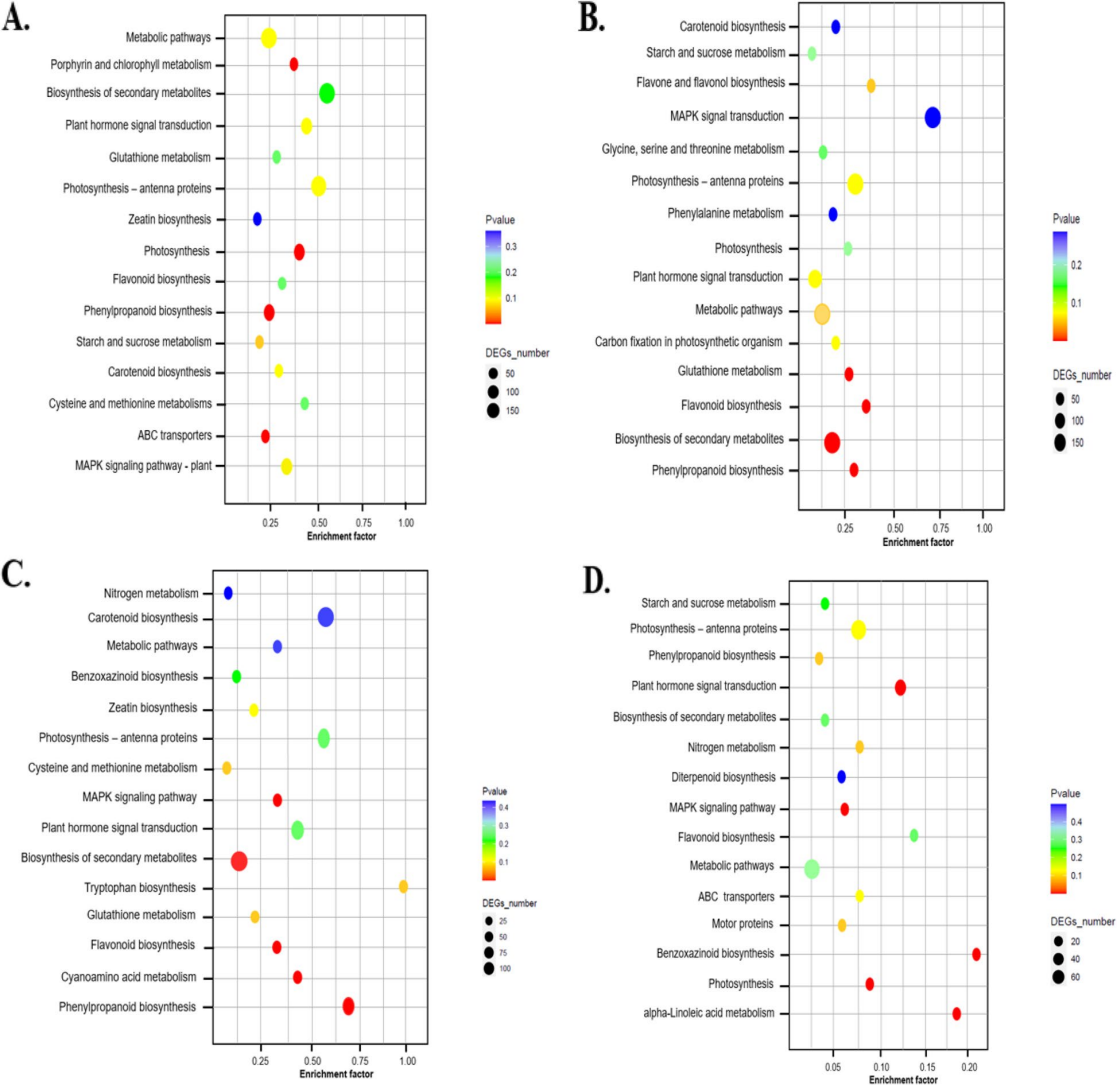
Kyoto Encyclopedia of Genes and Genome (KEGG) enrichment of differentially expressed genes (DEGs) under CF vs NA (**A**), SC vs NA (**B**), SM vs NA (**C**), and MS vs NA (**D**). Top 15 KEGG enrichment pathways among the fertilization genotypes based on annotation information. NA, No fertilization; CF, inorganic fertilizer; SC, inorganic fertilizer plus commercial organic fertilizer; SC, commercial organic fertilizer; MS, maize straw

To further investigate the biological functions in maize leaf tissue in response to fertilization treatments, DEGs from CF vs. NA, SC vs. NA, SM vs. NA, and MS vs. NA comparisons were analyzed against the KEGG pathway database. The analysis revealed that 270 DEGs were enriched in 94 pathways for CF vs. NA, 176 DEGs in 69 pathways for MS vs. NA, 301 DEGs in 102 pathways for SC vs. NA, and 200 DEGs in 90 pathways for SM vs. NA (Fig. 2A-D; Supplementary Table S4). In the CF vs. NA treatment, biosynthesis of secondary metabolites and photosynthesis pathways were up-regulated, whereas metabolic pathways were downregulated (Fig. 2A; Supplementary Table S4). For the MS vs. NA treatment, pathways such as photosynthesis-antenna proteins, plant hormone signal transduction, and metabolic pathways showed up-regulated DEGs (Fig. 2B; Supplementary Table S4). In the SC vs. NA treatment, the MAPK signal transduction and metabolic pathways were notably downregulated, while photosynthesis-antenna proteins and biosynthesis of secondary metabolites had up-regulated genes (Fig. 2C; Supplementary Table S4). Additionally, genes involved in carotenoid biosynthesis, phenylpropanoid biosynthesis, biosynthesis of secondary metabolites, and photosynthesis-antenna proteins pathways were significantly enriched in the SM vs. NA treatment relative to SC vs. NA, CF vs. NA and MS vs. NA (Fig. 2D; Supplementary Table S4).

### Identification of DEGs related to transcription factors genes in response to fertilization

**Fig. 3:**
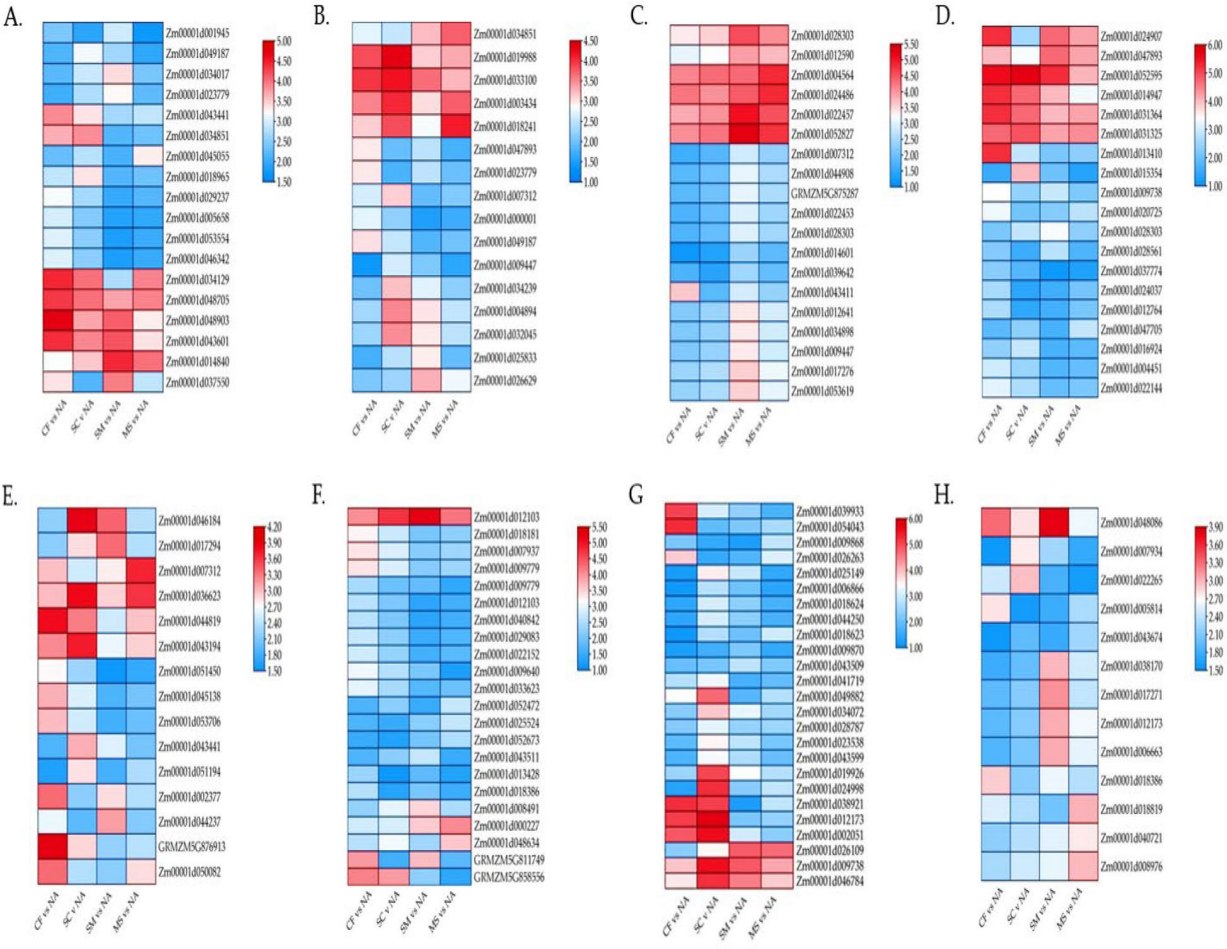
Identification of differentially expressed genes (DEGs) associated with transcription factors (TFs) in reaction to fertilization. The heat map displays the relative expression of TF family DEGs, including (**A**) NAC, (**B**) MYB, (**C**) FARI, (**D**) ERF, (**E**) BHLH, (**F**) B3, (**G**) HB, and (**H**) WRKY. The colour gradient from blue to red represents the range from low to high relative expression levels

Transcription factors (TFs) play crucial roles in leaf tissue growth by promoting cell division and modulating physiological processes or responses to stress. In this study, we identified a total of 3,530 TF genes across 45 TF families. The most commonly identified DEGs encoded members were the NAC, MYB, FARI, ERF, bHLH, B3, HB-other, and WRKY families (Fig. 3 A-H; Supplementary Table S5). The NAC family, which is highly responsive to abiotic stress and fertilization, included 346 DEGs. Additionally, there were 338, 289, 269, 266, 253, 255, and 172 DEGs belonging to the MYB, FARI, ERF, bHLH, B3, HB-other, and WRKY families, respectively (Fig. 3 A-H). Notably, in the NAC TF family, four genes (Zm00001d046784, Zm00001d009738, Zm00001d002051, and Zm00001d012173) showed upregulation predominantly in the **SC** vs. NA comparison. Furthermore, NAC (Zm00001d054043) and FARI (Zm00001d024907 and Zm00001d013410) genes were downregulated, particularly in the CF vs. NA comparison. Overall, the gene expression patterns varied, with significant up- and down-regulation in the MS vs. NA and SM vs. NA comparisons (Fig. 3 A-H; Supplementary Table S5). Therefore, the distinct expression patterns observed under SC vs. NA and CF vs. NA treatments compared to SM vs. NA and MS vs. NA may be critical for the regulatory mechanisms of TF families in maize leaf tissue.

### Impact of fertilization of DEGs related to photosynthesis

Fertilization practices have a significant impact on the photosynthetic capacity of leaf tissue, which plays a crucial role in the development of kernels in maize plants. Our study identified several genes expressed in various components of photosynthesis, including photosynthesis-antenna protein, photosynthesis complex II, photosynthesis complex I, cytochrome complex, electron transport, and ATPase complex I, which was markedly affected by CF vs. NA, SC vs. NA, SM vs. NA, and MS vs. NA treatment comparisons (Supplementary Table S6).

A total of 19 genes were expressed in the photosynthesis-antenna protein category in response to fertilization treatments. In the CF vs. NA treatment, two genes (Zm00001d044396 and GRMZM2G429955) related to the light-harvesting complex were significantly up-regulated. In contrast, in the SM vs. NA treatment comparison, one gene (Zm00001d011285) was notably downregulated (Supplementary Table S4). The genes encoding photosynthesis complex II comprised 28 DEGs, derived from the photosystem family protein and reaction center. The expression level of photosystem complex II increased by 1.64 to 2.31 folds under the SC vs. NA treatment compared to the CF vs. NA, SM vs. NA, and MS vs. NA treatment comparisons (Supplementary Table S6).

Nineteen genes were expressed in photosynthesis complex I, with the majority being downregulated. However, high expression levels were observed in the SM vs. NA and MS vs. NA treatment comparisons, particularly in genes related to the photosystem I reaction and ferredoxin-NADP ( +)-oxidoreductase. Two genes involved in the cytochrome complex were derived from photosynthetic electron transfer (PETC and PGR1). Notably, one gene (Zm00001d053432) was up-regulated under SC vs. NA and MS vs. NA treatment, showing a 0.52-fold higher expression relative to CF vs. NA and SM vs. NA treatment comparisons (Supplementary Table S6).

Thirteen genes related to photosynthetic electron transport were identified, consisting of seven genes encoding ferredoxin superfamily protein (FED) and six genes encoding photosynthetic electron transfer (PETE). Among these, three genes (Zm00001d012293, GRMZM5G800780, and GRMZM5G884960) exhibited significantly higher expression levels in the CF vs NA treatment, showing upregulation compared to other fertilization treatments (Supplementary Table S6). Additionally, five genes encoding ATPase complex I were identified, comprising three genes in the ATP synthase delta-subunit gene (ATPD) and two genes in the ATPase F1 complex gamma subunit protein (ATPC1). The expression of the ATPD gene (Zm00001d033922) was downregulated in the SC vs NA treatment but significantly higher compared to CF vs NA, SM vs NA, and MS vs NA treatments (Supplementary Table S6).

### Fertilization-induced changes in DEGs involved in phytohormone in maize

Phytohormones play a crucial role in Maize's response to abiotic stress and fertilization. We identified several phytohormone components in maize leaf tissue, including auxin (AUX), cytokinins (CTK), gibberellin (GA), abscisic acid (ABA), ethylene (ETH), brassinosteroid (BR), jasmonic acid (JA), and salicylic acid (SA) (Figs. 5 and 6).

**Fig. 4:**
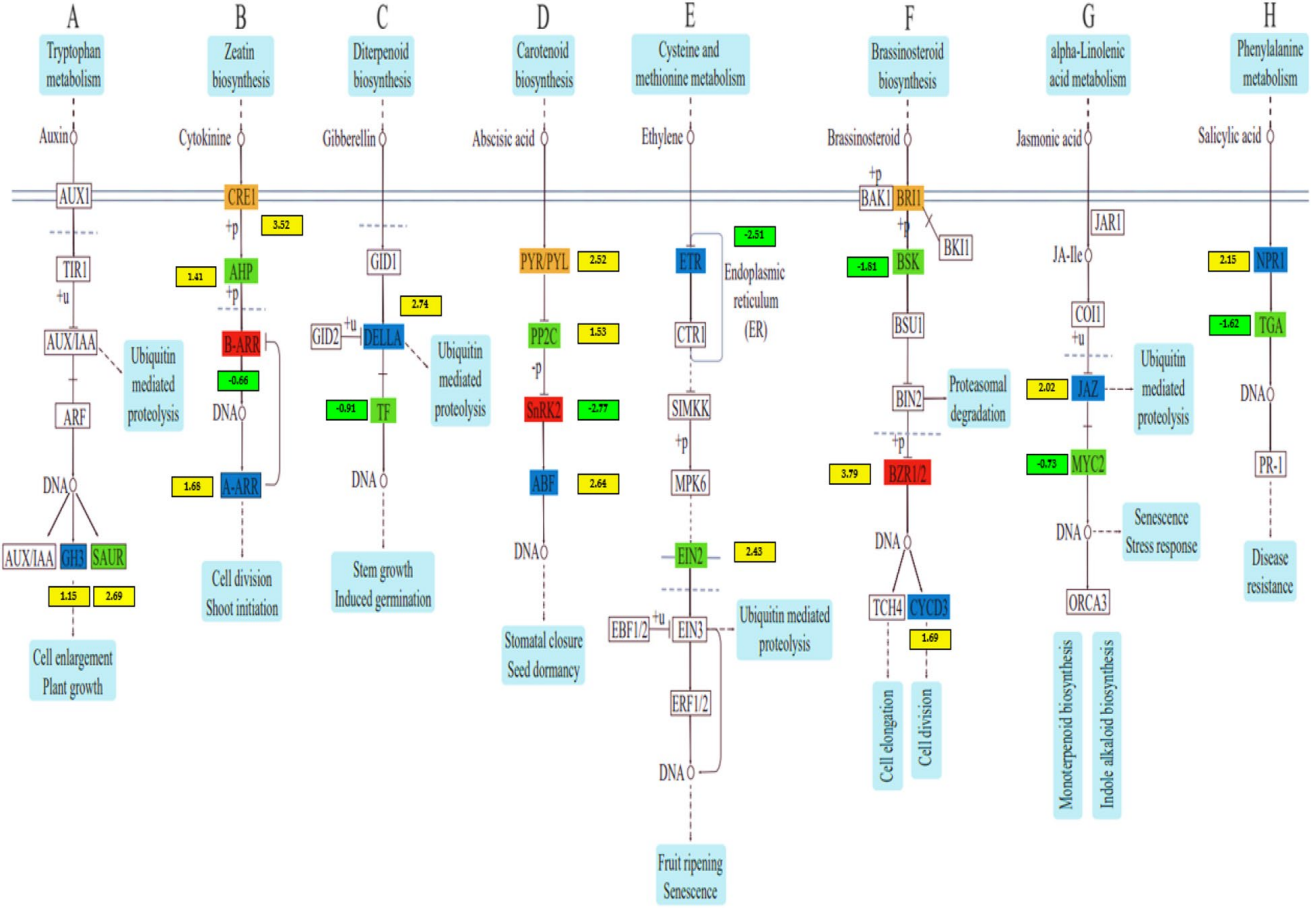
DEGs involved in plant hormone signaling pathway summary of the maize plant response under fertilization. **A** Auxin (**B**), Cytokinin (**C**), Gibberellin (**D**) Abscisic acid, (**E**) Ethylene, (**F**) Brassinosteroid (**G**) Jasmonic acid, and (**H**) Salicylic acid signaling pathway. The absolute values of log2 ≥ 1 and FDR < 0.001 were used as the criteria for DEGs. The colour of the box had key enzymes, it also represents up (yellow) and down (green)-regulated genes, and the value in the box is the log2 of the genes in the ear leaf under fertilization

**Fig. 5:**
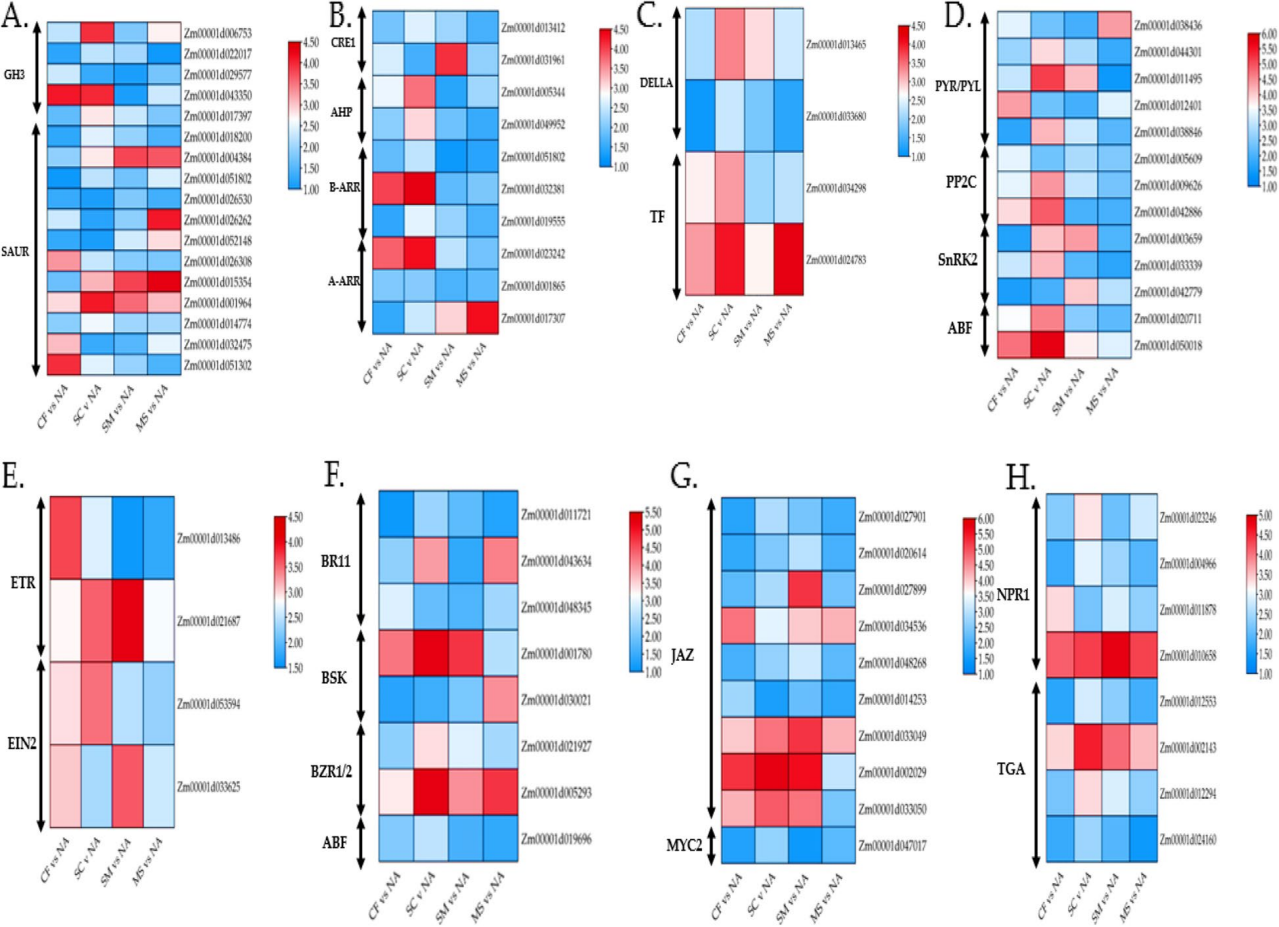
Heatmap of DEGs within vital enzymes involved in hormone and signal transduction pathways in two pairwise comparisons under fertilization. **A** Auxin (**B**), Cytokinin (**C**), Gibberellin (**D**) Abscisic acid, (**E**) Ethylene, (**F**) Brassinosteroid (**G**) Jasmonic acid, and (**H**) Salicylic acid signaling pathway. The colour gradient from blue to red represents the range from low to high relative expression levels

**Fig. 6:**
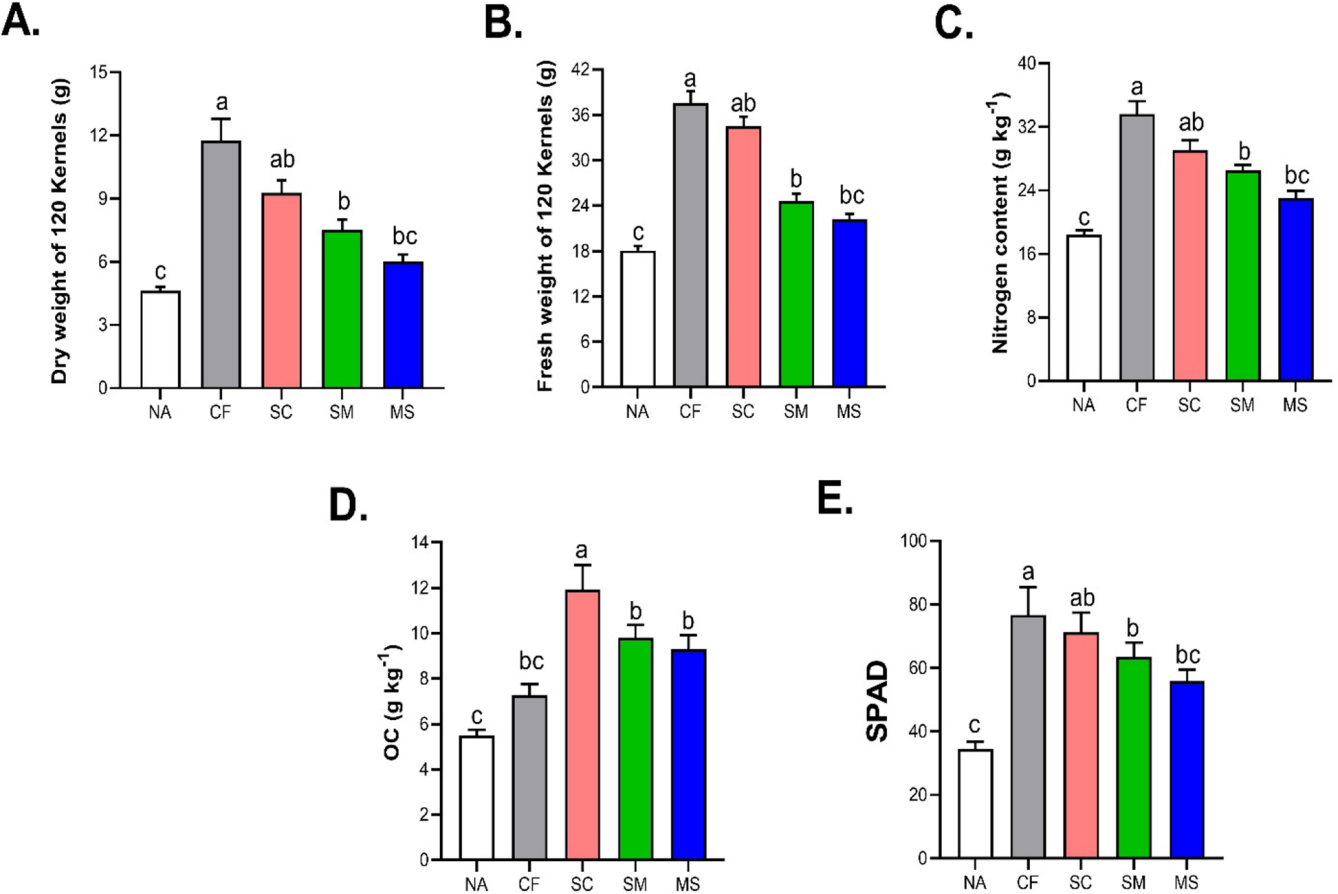
Effects of Fertilization on Grain dry and fresh weight (**A**, **B**), Nitrogen and Organic Carbon content (**C**, **D**) and (**E**) SPAD. Values with different letters indicate significant differences among treatments, with error bars that represent the standard deviation (*P* < 0.05, Duncan multiple-range test). NA, No fertilization; CF, inorganic fertilizer; SC, inorganic fertilizer plus commercial organic fertilizer; SC, commercial organic fertilizer; MS, maize straw

In the auxin signaling pathway, four GH3 (auxin-responsive GH3 gene family) genes and twelve SAUR (small auxin RNA) genes showed up-regulated expression (Figs. 4 and 5A). One GH3 gene (Zm00001d043350) was significantly expressed in both SC vs. NA and CF vs. NA treatments, while another gene (Zm00001d006753) was dominant only in the SC vs. NA relative to other treatments. Among the SAUR genes, two (Zm00001d026262 and Zm00001d015354) were highly expressed in the MS vs. NA treatment, and one gene (Zm00001d0011964) showed higher expression in the SC vs. NA treatment (Figs. 5 and 6A). For the cytokinin pathway, two genes (Zm00001d013412 and Zm00001d031961) encoding cytokinin-dependent receptors (CRE1) were up-regulated, along with two genes encoding histidine-containing phosphotransferase (AHP). Three genes encoding B Arabidopsis response regulators (B-ARR) were downregulated. In comparison, three genes encoding A Arabidopsis response regulator (A-ARR) showed differential regulation, with two genes (Zm00001d023242 and Zm00001d017307) being regulated in the SC vs. NA and MS vs. NA treatments, respectively, relative to other treatments. These cytokinin-binding receptors transduce signals through the plasma membrane in leaf tissue (Figs. 4 and 5B).

In the gibberellin pathway, two genes in the DELLA family were up-regulated. In contrast, two transcription factor (TF) genes (Zm00001d034298 and Zm00001d024783) were downregulated, showing dominance in the SC vs. NA and MS vs. NA comparisons (Figs. 4 and 5C). The abscisic acid (ABA) pathway revealed that five PYR/PYL genes (Zm00001d038436, Zm00001d044301, Zm00001d011495, and Zm00001d012401) were up-regulated, particularly in the SC vs. NA treatment. Additionally, three protein phosphatase 2C (PP2C) genes were up-regulated, while three serine/threonine-protein kinase SRK2 (SnRK2) genes (Zm00001d003659, Zm00001d033339, and Zm00001d042779) were downregulated. Two ABA-responsive element binding factor (ABF) genes (Zm00001d020711 and Zm00001d050018) were up-regulated in the CF vs. NA and SC vs. NA treatments (Figs. 4 and 5D). In the ethylene pathway, two ethylene receptor (ETR) genes (Zm00001d013486 and Zm00001d021687) were downregulated, particularly in the SM vs NA treatment. Conversely, two ethylene-insensitive proteins 3 (EIN2) genes (Zm00001d053594 and Zm00001d033625) showed upregulation in the CF vs. NA, SC vs. NA, and SM vs. NA treatments (Figs. 4 and 5E). For the brassinosteroid pathway, three BRI1 kinase inhibitor 1 (BKI1) genes were downregulated. At the same time, two brassinosteroid signaling kinase (BSK) genes (Zm00001d001780 and Zm00001d030021) were also downregulated, showing dominance in CF vs. NA, SC vs. NA, and SM vs. NA. Additionally, two genes (Zm00001d005293 and Zm00001d021927) linked to brassinozole-resistant 1 and 2 (BZR1/2) were up-regulated across all fertilization treatments. One gene related to cyclin-dependent kinase D3 (CYCD3) was identified (Figs. 4 and 5F).

In the jasmonic acid pathway, nine jasmonate zim-domain protein (JAZ) genes were up-regulated, with two genes (Zm00001d002029 and Zm00001d033049) showing increased expression in SC vs. NA and SM vs. NA compared to MS vs. NA and CF vs. NA. Only one MYC-type 2 transcription factor (MYC2) gene (Zm00001d047017) was downregulated (Figs. 4 and 5G). In the salicylic acid pathway, four non-expression of pathogenesis-related genes 1 (NPR1) genes were up-regulated, with Zm00001d010658 showing dominance in SM vs NA compared to CF vs. NA, SC vs. NA, and MS vs. NA. Additionally, four TGACG sequence-binding factor (TGA) genes were downregulated, with higher expression in SC vs. NA compared to CF vs. NA, SM vs. NA, and MS vs. NA (Figs. 4 and 5H).

These findings indicate that endogenous phytohormone pathways involving AUX, CTK, ABA, ETH, BR, JA, and SA play significant roles in maize leaf tissue's response to fertilization treatments (CF vs. NA, SC vs. NA, SM vs. NA, and MS vs. NA), in contrast to the GA pathway.

### Morphological features of maize leaves under fertilization treatments

Fertilization practices significantly impacted grain dry and fresh weight yield, nitrogen content, Soil organic carbon (SOC) and Chlorophyll content during the growing seasons, with notable differences among the fertilization treatments (CF, SC, SM, and MS) (Fig. 6A-E). The grain dry weight yield increased by 48.73% in CF and 40.05% in SC treatments compared to the NA treatment (Fig. 6A). On average, fresh weight yield increased by 2.25, 2.15, 1.36, and 1.31 fold in CF, SC, MS, and SM treatments, respectively, compared to the NA treatment (Fig. 6B). Additionally, the nitrogen content in leaves significantly increased (*p* < 0.05) in CF and SC treatments compared to the NA treatment; however, SC dominated OC concentration (Fig. 6 C-D).

The leaf chlorophyll content in Maize, measured by the SPAD value, increased with the application of various fertilization practices (Fig. 1D). The SPAD values for the CF, SC, SM, and MS treatments were 33.71%, 47.24%, 50.36%, and 53.81% higher, respectively, compared to the NA treatment. However, there was no significant difference between the CF and SC treatments (Fig. 6E).

**Fig. 7:**
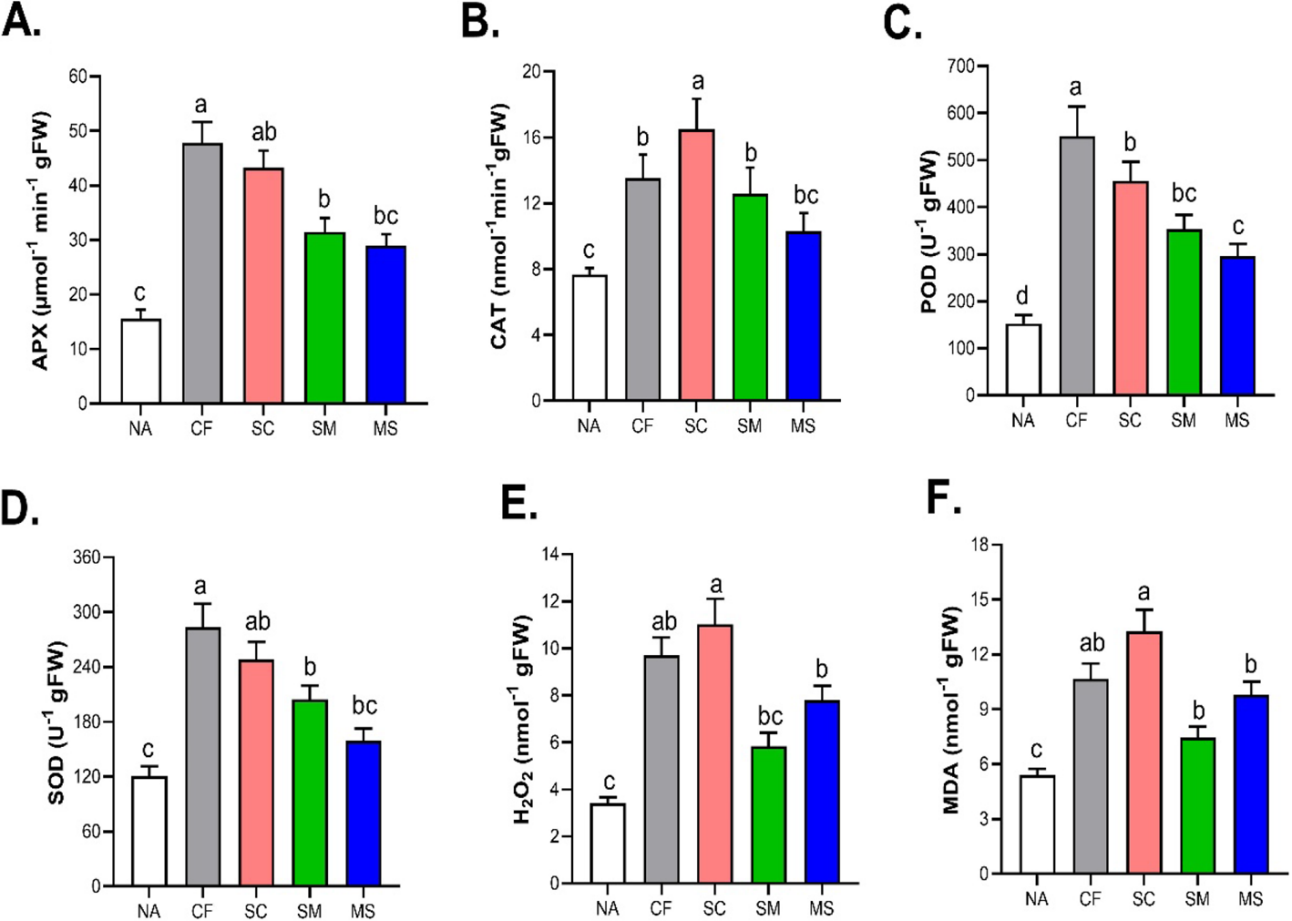
Effects of fertilization on the antioxidant enzyme activity (**A**), APX (**B**), CAT (**C**), POD (**D**) SOD, as well as the Physiological effects of reactive oxygen species (ROS) and MDA contents (**E**–**F**). The data shown are the means of three replicates (± SD) based on Duncan's multiple-range test. Means denoted with the same letter did not significantly differ at *p* < 0.05. Ascorbate peroxidase (APX), Catalase (CAT), Peroxidase (POD), Superoxide dismutase (SOD), Malondialdehyde (MDA), and Hydrogen peroxide (H_2_O_2_). NA, No fertilization; CF, inorganic fertilizer; SC, inorganic fertilizer plus commercial organic fertilizer; SC, commercial organic fertilizer; MS, maize straw

We measured the activities of reactive oxygen species (ROS), particularly Hydrogen peroxide (H_2_O_2_) and malondialdehyde (MDA), as well as antioxidant enzymes, (Ascorbate (APX), Catalase (CAT), Peroxidase (POD) and superoxide dismutase (SOD) activity content under fertilization (NA, CF, SC, SM and MS) treatments. The activity levels of APX and POD increased in CF, SC, SM and MS by (25.76%, 29.4%, 32.6% and 37.4%) and (28.4%, 31.1%, 34.2% and 39.6%) relative to NA treatment (Fig. 7A and C). The activity of CAT with SC was significantly higher followed by CF, SM and MS treatments compared to NA treatment (Fig. 7B). However, the SOD content increased significantly under SC, CF, SM and MS treatments by 3.32, 3.24, 2.15, and 1.94 fold compared to NA treatment (Fig. 7D). The H_2_O_2_ and MDA contents of SC and CF treatments were significantly increased by 36.4% and 39.2% relative to NA treatment (Fig. 7E and F).

### Validation of DEGs by qRT-PCR

**Fig. 8:**
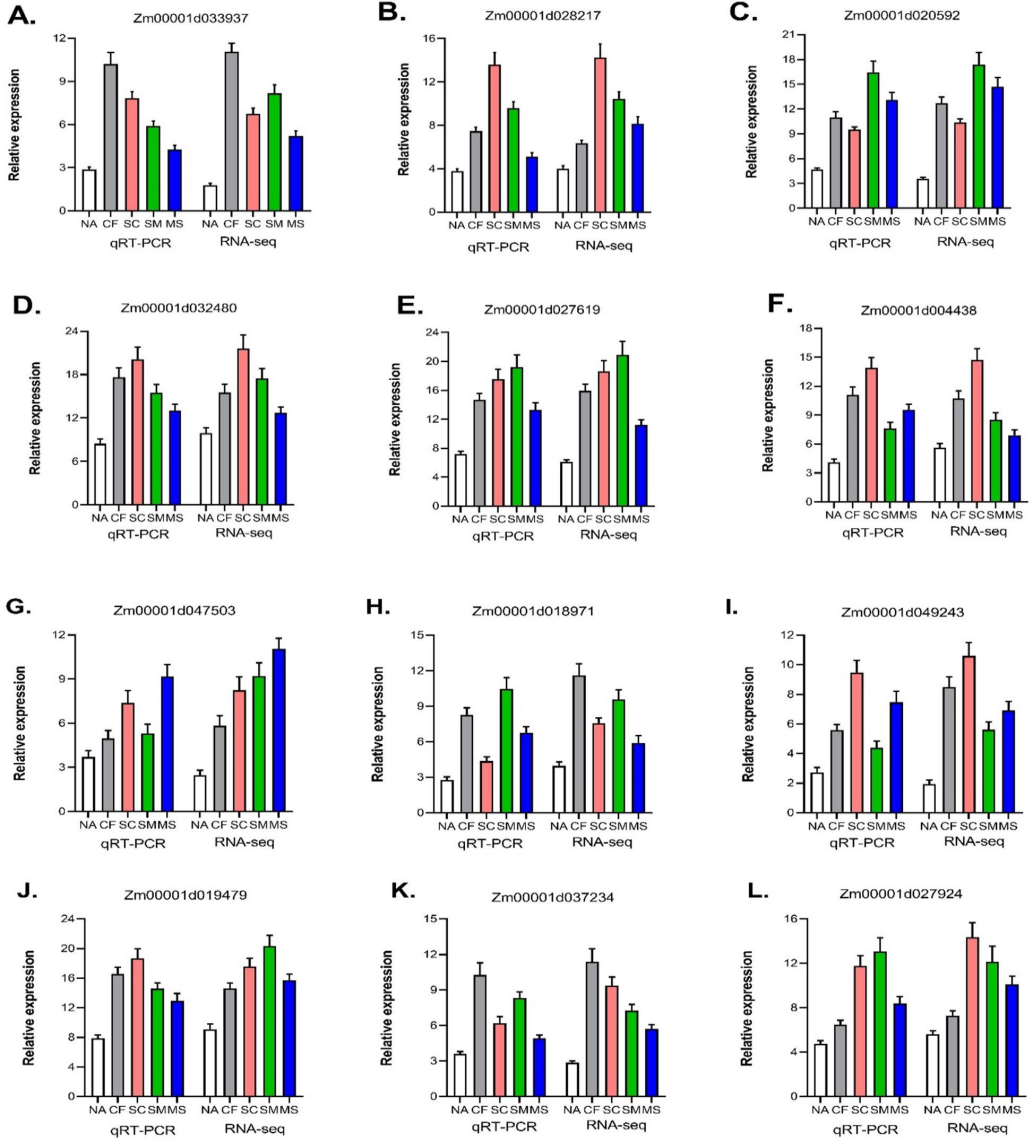
Senescence-dependent changes in gene expression determined by Real-time quantitative PCR (qRT-PCR) validation of RNA-Seq analysis in maize grains under fertilization. Different lowercase letters denote significant differences at *P* < 0.05

To confirm the expression patterns of DEGs identified by RNA-Seq analysis, the expression levels of twelve selected DEGs were examined using qRT-PCR under various fertilization treatments (NA, CF, SC, SM, and MS) (Fig. 8A-L). Despite minor differences in expression levels, the qRT-PCR results for nearly all twelve genes showed patterns consistent with the RNA-Seq analysis, validating the RNA-Seq findings. Pearson correlation analysis between the gene expression levels measured by qRT-PCR and RNA-Seq showed a significant correlation (correlation coefficient, *R* = 0.913), supporting the reliability of the sequencing data (Supplementary Fig. S4).

## Discussions

Excessive use of synthetic nitrogen fertilizers can cause environmental harm through nitrate leaching, runoff, and water pollution. A balanced nutrient supply, including nitrogen, potassium, and phosphorus, is essential for maize growth and yield. Inadequate nutrients negatively impact plant physiology and crop performance in the semiarid regions [35, 36]. Leaves, which have a consistent developmental history, are ideal for studying plant development. Throughout their growth, leaves undergo various developmental, physiological, and metabolic changes, ultimately leading to senescence and death. Leaf senescence is crucial for grain filling and biomass production, highlighting the importance of effective fertilization management. Combining chemical and organic fertilizers enhances nitrogen and soil organic carbon content, leading to increased grain yield and better nutrient synchronization, resulting in higher yields and moderate nutrient use efficiency [37–39].

Fertilization practices greatly affect plant gene expression, microbiome composition, and transcriptome profiles, leading to biochemical and physiological changes. Transcriptome analysis helps identify key genes involved in leaf senescence, photosynthesis, and transcription factors [19]. In this study, 8,161 differentially expressed genes (DEGs) were identified across various fertilization treatments. Most DEGs were found in the comparison of chemical fertilizer (CF) versus no application (NA), indicating that combining inorganic (CF) with organic fertilizers (SC) significantly influences nutrient accumulation and remobilization in Maize [10]. Conversely, the absence of fertilization suppressed gene expression more than the application of maize straw (MS) and sole organic fertilizer (SM).

To further investigate the transcriptional functions of how maize leaf tissue responds to different fertilization treatments, a KEGG pathway analysis of differentially expressed genes (DEGs) was conducted. The analysis showed significant enrichment in pathways related to photosynthesis-antenna proteins, plant hormone signal transduction, and secondary metabolites. Phytohormones, which are key regulators of plant senescence and development, were notably involved. Ethylene (ETH), jasmonic acid (JA), salicylic acid (SA), and abscisic acid (ABA) promote senescence, while cytokinins (CTK), auxins (AUX), and gibberellins (GA) delay it [2, 40].

ABA, a crucial phytohormone, mediates plant responses to abiotic stresses and plays a significant role in leaf senescence under fertilization conditions. In this study, ABA receptor components such as PYR/PYL, PP2C, HAB, ABFs, and SnRK2 were differentially expressed in leaves subjected to inorganic and organic fertilization treatments (Figs. 5 and 6). PYR/PYL genes were highly expressed in SC vs. NA treatments, while PP2C, HAB, ABFs, and SnRK2 showed higher expression in CF vs. NA, SC vs. NA, and SM vs. NA treatments. During leaf senescence, ABA levels rise, with PYR/PYL receptors detecting this change and activating downstream signaling pathways. PP2C proteins negatively regulate ABA signaling by dephosphorylating SnRK2 kinases in the absence of ABA, but during senescence, ABA binding inhibits PP2C activity, allowing SnRK2 activation [40, 41]. HAB proteins may influence senescence-related processes through their interaction with ABA pathways. ABFs, activated by SnRK2 kinases, regulate the expression of ABA-responsive genes involved in leaf senescence. SnRK2 kinases, upon ABA perception and PP2C inhibition, phosphorylate downstream targets, including ABFs, initiating gene expression involved in senescence. Overall, the combined effect of inorganic and organic fertilization impacts ABA signaling pathways, altering the activity of PYR/PYL receptors, PP2C, HAB, ABFs, and SnRK2, thereby influencing leaf senescence regulation [42, 43].

Ethylene, a key plant hormone synthesized from methionine, is crucial for regulating leaf senescence, and its interaction with fertilization influences the expression of senescence-promoting genes in maize leaves [43, 44]. Ethylene is perceived by membrane-bound receptors, activating downstream signaling components like EIN2 (ethylene insensitive 2), ethylene receptor (ETR), and EIN3/EIL (ethylene insensitive 3/ethylene insensitive-like) transcription factors. Fertilization can modulate ethylene signaling by affecting these components' expression or activity. In this study, two ETR genes (Zm00001d013486 and Zm00001d021687) were prominent under SM vs. NA treatment, while three EIN2 genes (Zm00001d053594 and Zm00001d033625) were notable in CF vs. NA, SC vs. NA, and SM vs. NA treatments (Figs. 5 and 6). EIN2 transmits ethylene signals to the nucleus, activating EIN3/EIL and ETR transcription factors, which regulate ethylene-responsive genes involved in leaf senescence. Ethylene controls genes associated with senescence, including those encoding senescence-associated proteins (SAGs), proteases, and nutrient remobilization enzymes [45, 46]. Fertilization influences these gene expressions either directly through ethylene signaling pathways or indirectly by altering nutrient availability or hormonal balance. The interaction between EIN2, ethylene receptors, and other signaling components under fertilization conditions ultimately determines the timing and progression of leaf senescence in response to environmental cues and nutrient availability [47, 48].

Jasmonate ZIM-domain proteins (JAZ) and Non-expression of Pathogenesis-Related Genes 1 (NPR1) are key components in the signaling pathways of jasmonic acid (JA) and salicylic acid (SA), respectively, and play significant roles in leaf senescence under different fertilization regimes. During senescence, rising JA levels lead to the degradation of JAZ proteins, activating MYC2 and other transcription factors to express senescence-related genes. NPR1, a major regulator of SA signaling, acts as a co-activator with TGA transcription factors to induce SA-responsive genes, including pathogenesis-related (PR) genes. The interactions between ETH, ABA, JA, SA, and other phytohormones are complex and context-dependent, influencing leaf senescence, plant defence, and stress responses under fertilization conditions [49–51]. Understanding these interactions and their synergistic effects is vital for optimizing fertilization practices to enhance crop productivity and stress tolerance.

Photosynthesis is vital for maize grain yield, influenced by stomatal and non-stomatal limitations like the structural integrity of photosynthetic protein complexes and enzyme activities. Leaf chlorophyll, crucial for photosynthesis, helps in light absorption, transport, and conversion [52, 53]. In this study, KEGG pathway analysis of differentially expressed genes (DEGs) under various fertilization treatments identified significant pathways (Supplementary Table 4). Specifically, key pathways included 19 genes in photosynthesis-antenna, 28 in photosystem II, 2 in the cytochrome complex, 19 in photosystem I, 13 in photosynthetic electron transport, and 5 in the ATPase complex, with more genes being up-regulated (Supplementary Table 6). Promoter analysis showed that these photosynthesis-related genes were regulated by ABA- and ETH-mediated stress response pathways. Up-regulated genes encoded structural proteins of photosystems, such as PETC, FED, PETE, and PGR1, directly enhancing photosynthesis [53]. The increased expression of these genes correlates with higher photosynthetic capacity, explaining the significant rise in photosynthetic rate and maize yield under combined inorganic (CF) and organic (SC) fertilization compared to no fertilizer (NA). Additionally, the gene atpB, related to ATP formation, was significantly up-regulated, aiding in converting light energy into ATP, further contributing to increased yield [54]. Therefore, enhanced photosynthetic capacity and energy metabolism in Maize are primary responses to fertilization. In this study, SC and CF treatments significantly increased SOD and CAT levels compared to NA, supporting enhanced activity of protective enzymes in leaves. Additionally, MDA accumulation in leaves may help regulate the balance between ROS production and elimination within cells. SOD, CAT, and POD are key ROS-scavenging enzymes in plants, and their activity reflects the plant's anti-aging capacity. MDA and H₂O₂ levels also serve as physiological and biochemical indicators of leaf senescence [2, 6].

Transcription factors (TFs) play a crucial role in regulating leaf senescence pathways by controlling the expression and activity of specific responsive genes in plants [55]. This study identified 3,530 TF genes across 45 TF families (Fig. 4; Supplementary Table S5). Key TF families, including NAC, MYB, FARI, ERF, bHLH, B3, HB-other, and WRKY, were found to be significant regulatory factors in plant responses to leaf senescence. Different members of these families exhibited varied expression patterns, with some being up-regulated and others down-regulated under different fertilization treatments. Notably, distinct expression patterns were observed under SC vs. NA and CF vs. NA treatments compared to SM vs. NA and MS vs. NA, which are important for the regulatory mechanisms in leaf senescence. Fertilization practices influence maize leaf senescence by affecting the expression of thousands of genes, including those controlling SAGs [56, 57]. Fertilization also alters the rhizosphere microbial community, indirectly affecting TF regulation involved in leaf senescence. Changes in nutrient status due to fertilization influence senescence-associated proteins and nutrient remobilization processes [45, 58]. TFs from the NAC, MYB, ERF, bHLH, HB-other, and WRKY families are essential components in the regulatory pathways of leaf senescence. Fertilization modulates the expression and activity of these key TFs, thereby affecting the downstream gene expression patterns that drive leaf senescence [58, 59].

In summary, fertilization affects the regulation of transcription factors such as NAC, MYB, ERF, bHLH, HB-other, and WRKY families, which are key to the complex regulatory networks governing leaf senescence. These networks influence processes like nutrient recycling, hormone signaling, programmed cell death, and the transcriptional control of senescence-associated genes [60, 61]. The qRT-PCR has been instrumental in exploring these networks, validating the expression of senescence-associated genes from transcriptome data, and understanding their regulatory roles [61]. Our research aims to identify sustainable fertilization practices that influence leaf senescence while enhancing maize grain yield and improving nitrogen use efficiency (NUE) in the semiarid Loess Plateau.

## Conclusions

Our study investigated how various fertilization methods impact the yield and molecular processes involved in maize leaf senescence and nutrient recycling. Across 8,161 differentially expressed genes (DEGs) by different fertilization treatments (CF, SC, SM, and MS), a higher proportion of genes were up-regulated than down-regulated. Molecular mechanisms revealed that the fertilizers influenced genes related to maize leaf senescence. Plants treated with organic fertilizers (MS and SM) and combined organic and inorganic fertilizer (SC) highly affected the DEGs related to photosynthesis-antenna proteins, plant hormone pathways, and metabolic pathways as compared to plants with inorganic fertilizers (CF). Increased antioxidant enzyme activity and improved photosynthesis work together to delay leaf senescence. Conversely, plants treated with inorganic fertilizers exhibited a higher number of DEGs related to ribosome pathways, biosynthesis of secondary metabolites, and photosynthesis pathways, resulting in increased protein content, 100-kernel weight, and yield. ABA receptor genes (PYR/PYL, PP2C, HAB, ABFs, SnRK2) and ethylene genes (EIN2, ETR, EIN3/EIL) were differentially expressed, particularly in leaves treated with inorganic (CF) and combined inorganic and organic (SC) fertilizers as compared to organic fertilizers alone (SM and MS). Fertilization also influenced key transcription factor families (NAC, MYB, FARI, ERF, bHLH, B3, HB-other, WRKY) that regulate maize plant responses to leaf senescence while highly up-regulated genes in the photosynthesis pathway encoded structural proteins of photosystems (PETC, FED, PETE, PGR1) across different fertilization treatments. The findings suggest strategies for breeders and farmers to reduce reliance on inorganic fertilizers while enhancing maize yields for food security. In future, isotope tracing could help to further elucidate DEG mechanisms across different maize genotypes, growth stages, and fertilizers.

## Supplementary Information

Supplementary Material 1.

Supplementary Material 2.

## Data Availability

All raw sequencing data from this study have been deposited at the NCBI under BioProject ID PRJNA1110623.
