# Supplementary material for: Gene expression through comparative transcriptome analysis unravels the molecular mechanisms of fertilizer-induced hormonal regulation and leaf senescence in maize for enhanced yield in semiarid regions
Source: BMC Plant Biol. 2025 Mar 13;25:327. doi: 10.1186/s12870-025-06303-5 (PMC11905492; doi:10.1186/s12870-025-06303-5)
Supplement: Supplementary file 2 — Supplementary Material 2. [file 12870_2025_6303_MOESM2_ESM.docx]

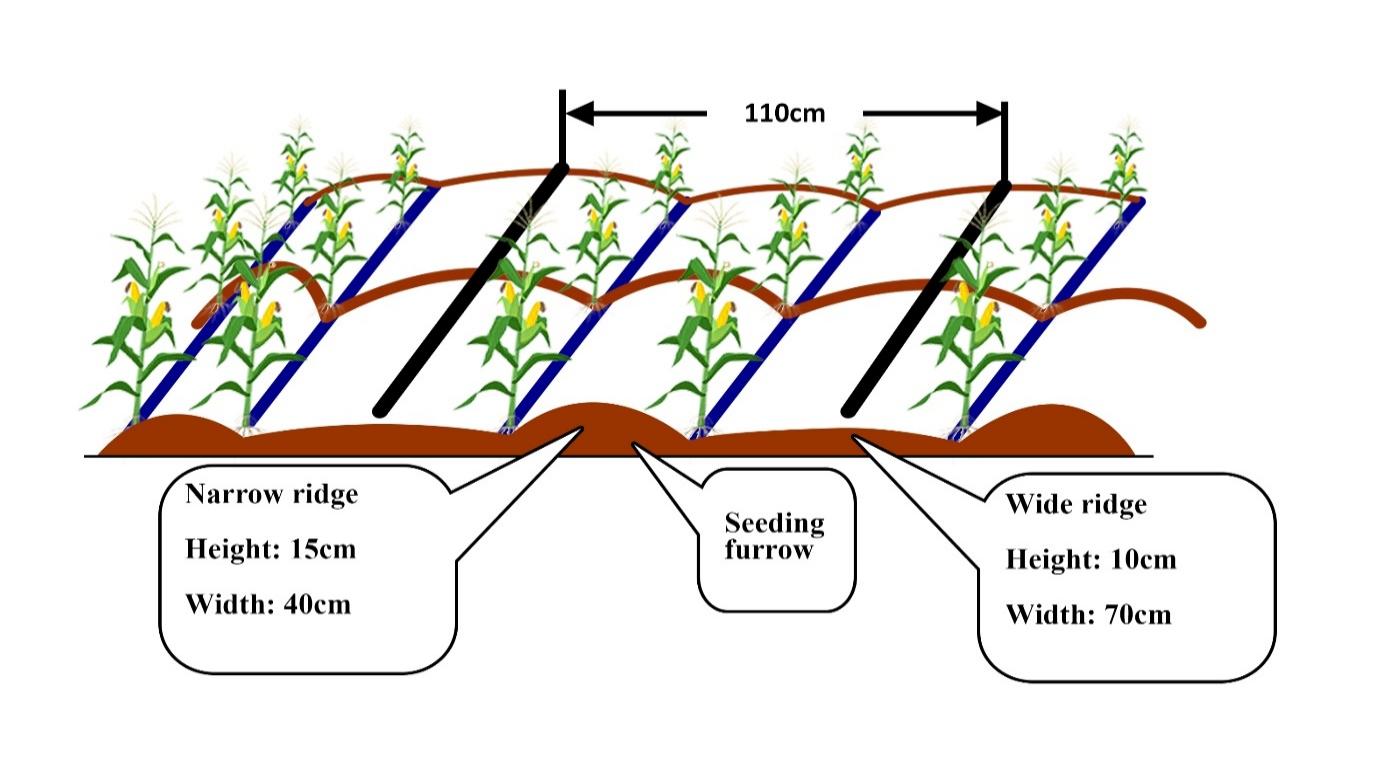


Supplementary Fig**.** S1. An overview of the plot showing ridges


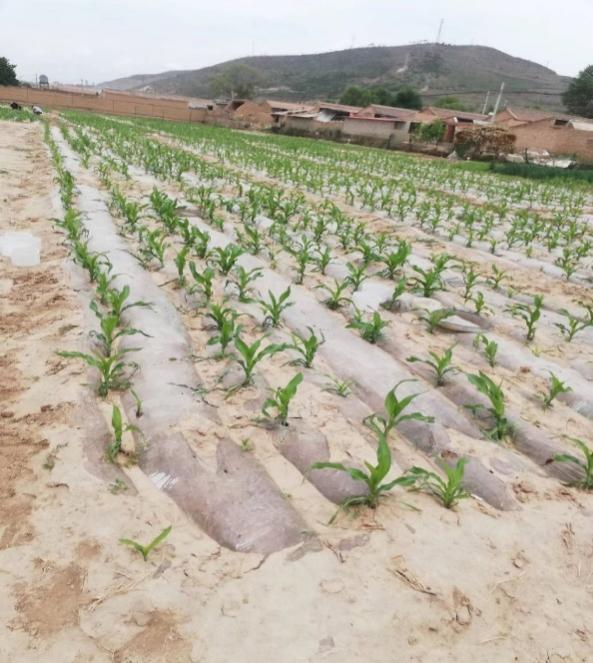

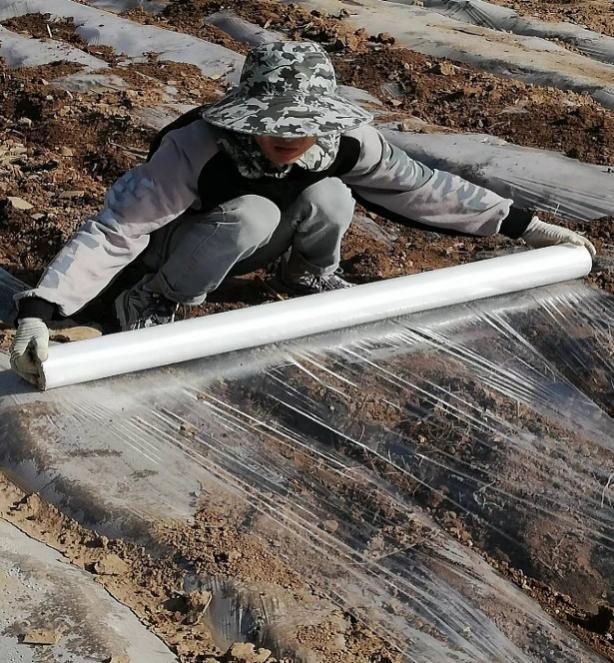


**(B)**

**(A)**

Supplementary Fig**.** S2**.** Diagram of (A-B) plastic film fully mulched ridge-furrow, in maize cropping system in the experiment for the study at the Rainfed Agricultural Experiment Station of Gansu Agricultural University.

Gene Ontology (GO)

CF vs NA


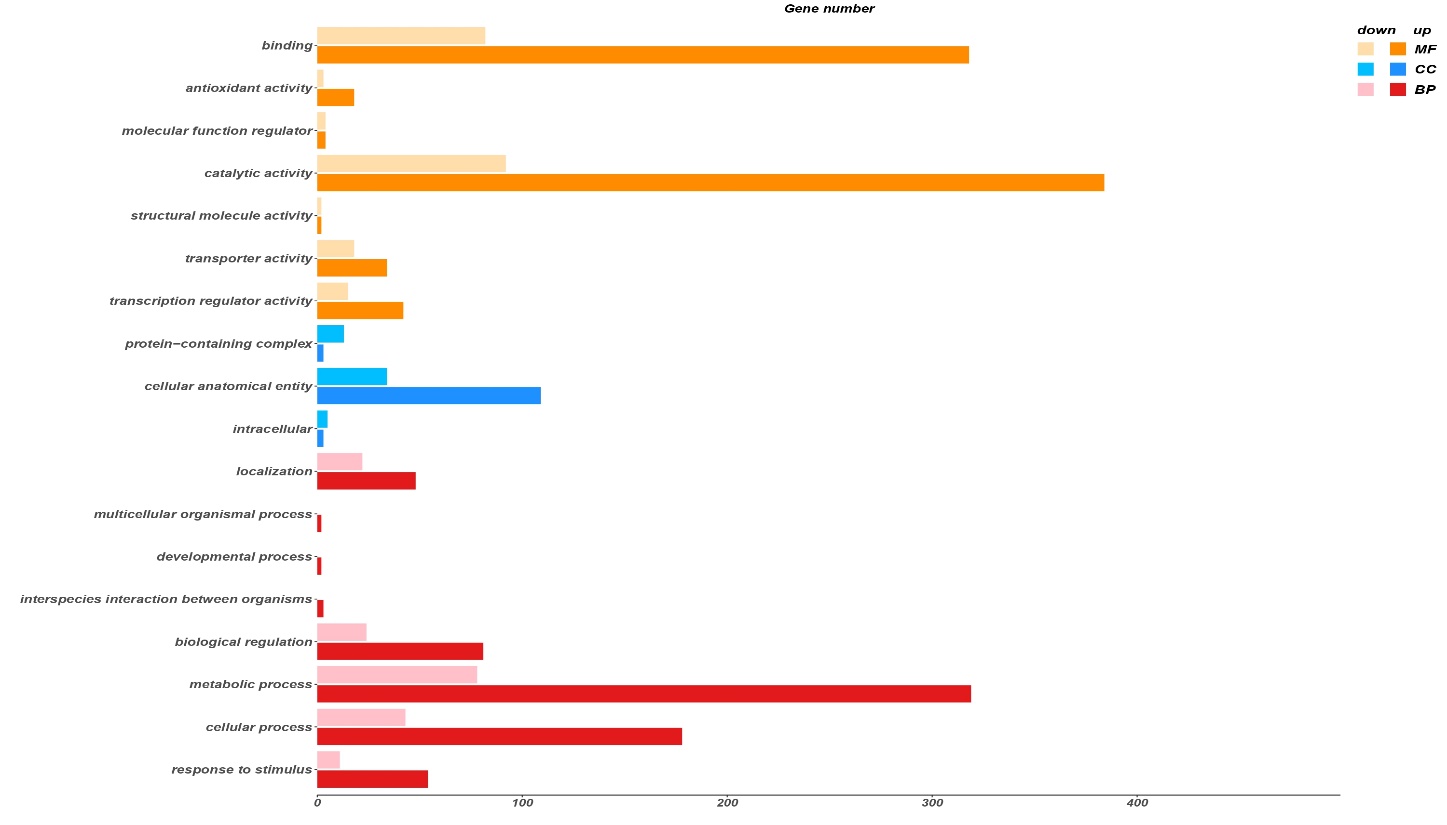


SC vs NA


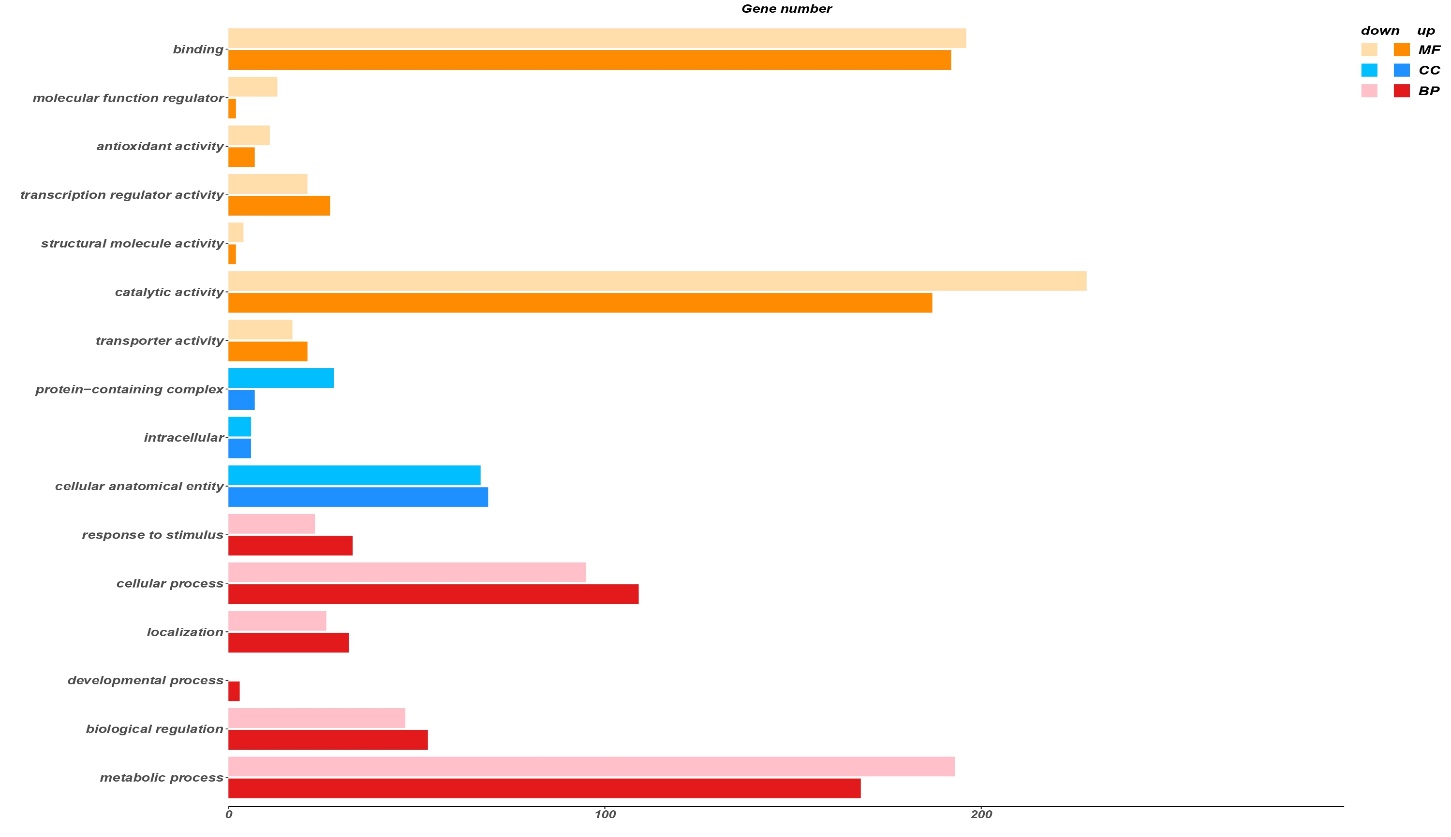


SM vs NA


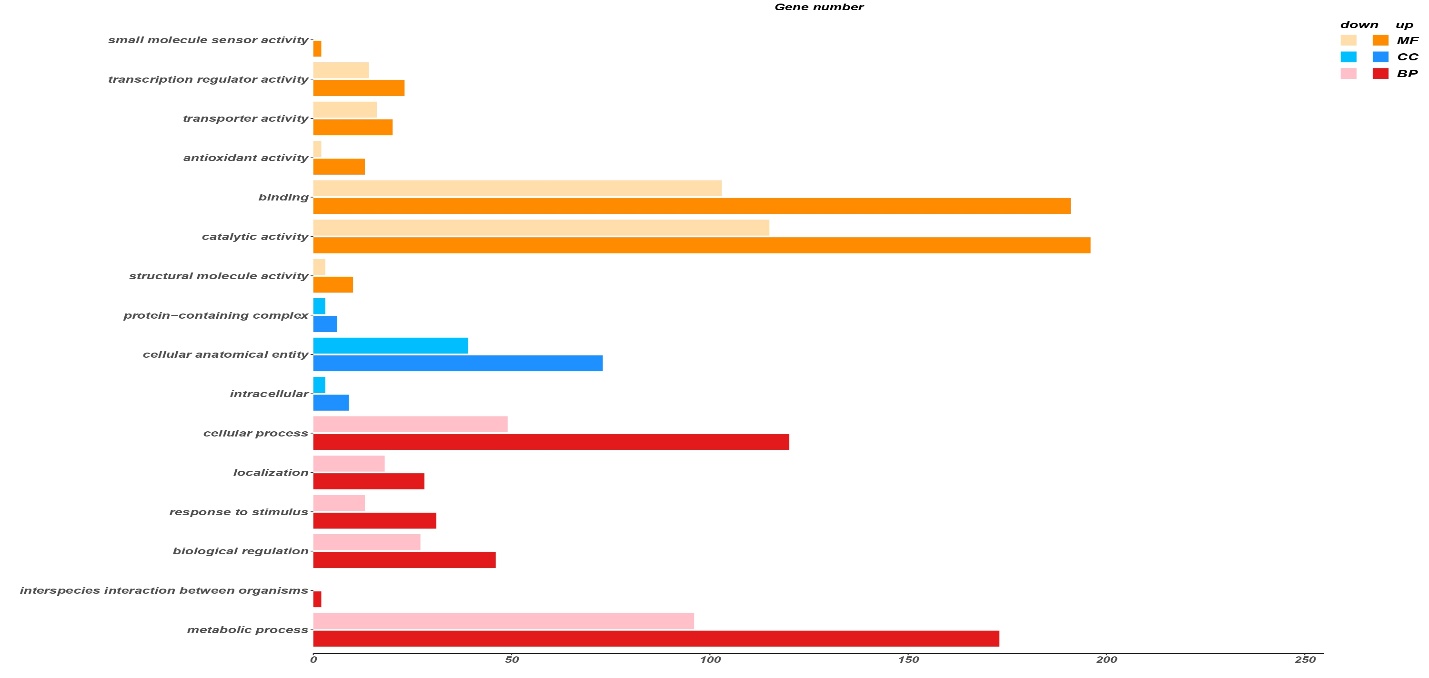


MS vs NA


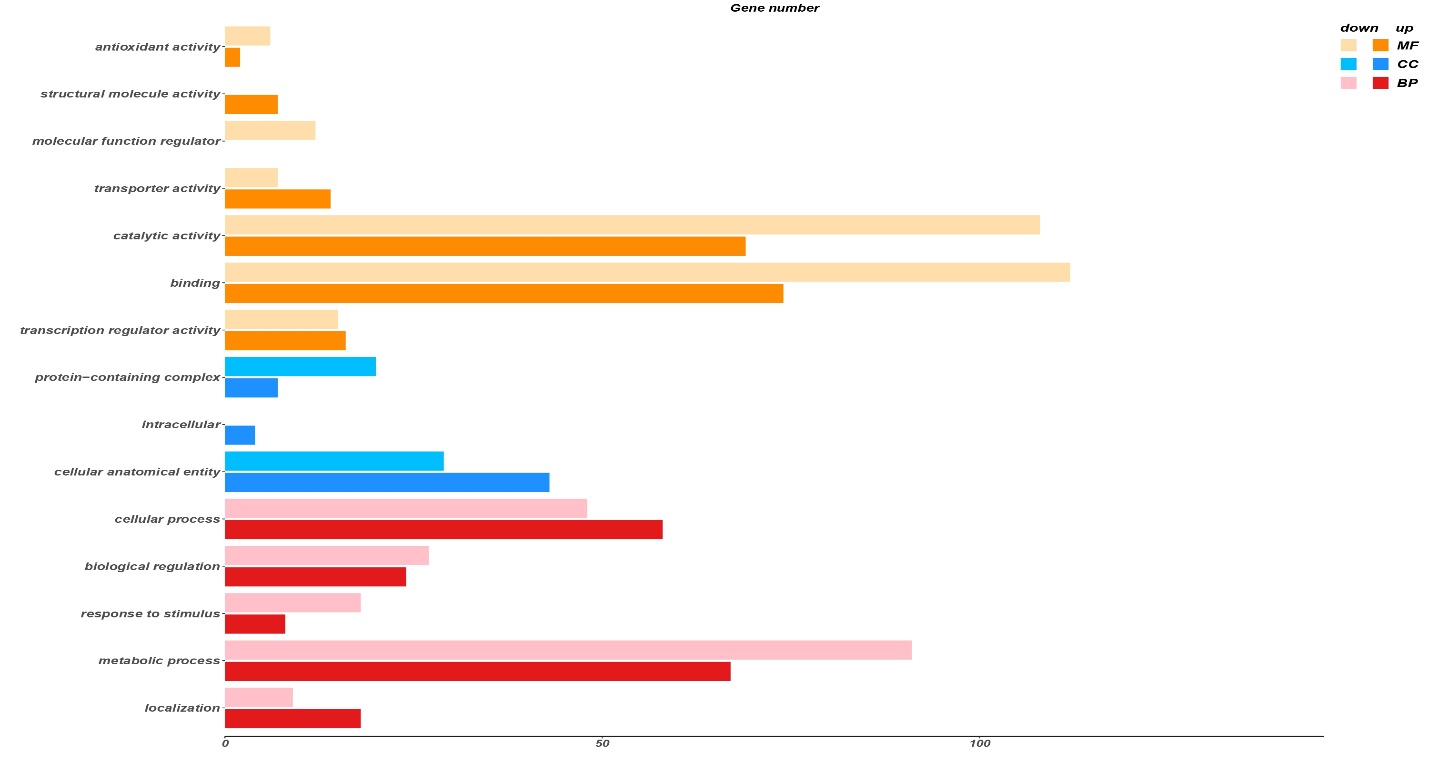


Supplementary Fig. S3. Gene-ontology (GO) functional-enrichment analysis of DEGs detected in maize leave tissue under CF vs NA (A), SC vs NA (B), SM vs NA (C), and MS vs NA (D). The Green columns indicate upregulated genes, and blue columns indicate downregulated genes. NA, No fertilization; CF, inorganic fertilizer; SC, inorganic fertilizer plus commercial organic fertilizer; SC, commercial organic fertilizer; MS, maize straw.


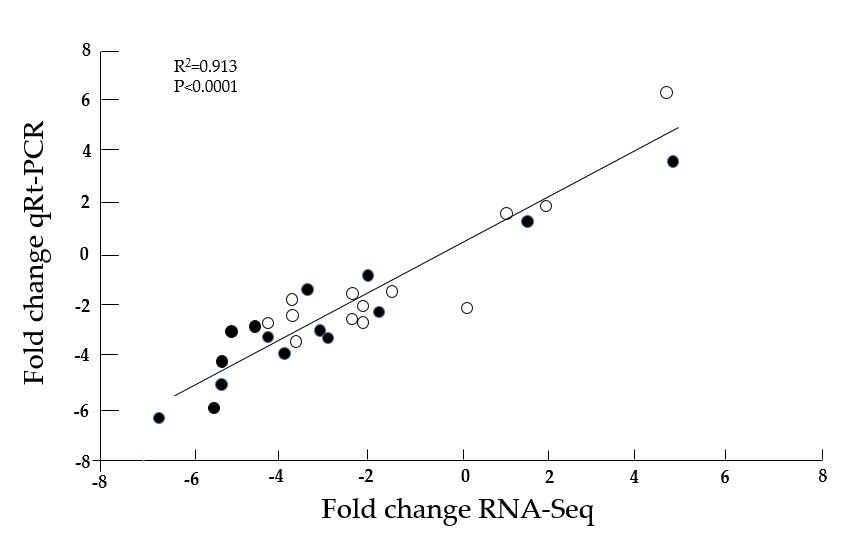


Supplementary Fig. S4. Pearson correlation analysis of gene-expression ratios obtained from qRT-PCR and RNA-Seq data. Error bars indicate standard error of the mean.
